# A meta-analysis of randomized control trials of surgical methods with osteosarcoma outcomes

**DOI:** 10.1186/s13018-016-0500-0

**Published:** 2017-01-13

**Authors:** Xiaojun He, Zhenzhen Gao, Hongwei Xu, Zhongwei Zhang, Peng Fu

**Affiliations:** 1 Department of Orthopedics, The Second Affiliated Hospital of Jiaxing College, 1518 Huancheng Rd, 314000 Jiaxing, China; 2 Department of Oncology, The Second Affiliated Hospital of Jiaxing College, Jiaxing, China

**Keywords:** Limb salvage surgery, Amputation, Local recurrence, 5-year overall survival, Meta-analysis

## Abstract

**Background:**

Osteosarcoma is a high malignant neoplasm, and conflicting findings have been reported on the survival and function recovery of osteosarcoma patients experiencing limb salvage or amputation. In the present study, we compared limb salvage surgery (LSS) with amputation in clinical outcomes of osteosarcoma patients by a meta-analysis.

**Methods:**

The survival rate of osteosarcoma patients was collected from research reports from CNKI, MEDLINE, EMBASE, the Cochrane Database, and Google Scholar till April 30, 2016. The quality of including articles was evaluated by two independent reviewers. Differences between patients undergoing limb salvage surgery and amputation were analyzed based on postoperative survival rates.

**Results:**

Ten articles were included according to selection criteria. There were 1343 patients in total from these studies. Our results showed that there was no significant difference between limb salvage surgery and amputation according to local recurrence; however, patients with limb salvage surgery had a higher 5-year overall survival.

**Conclusions:**

LSS results in higher 5-year survival rates and better survival, while not increasing the risk of local recurrence. This study provided more evidences to support limb salvage surgery as a considerable treatment of osteosarcoma patients.

## Background

Osteosarcoma is one rare kind of malignant tumors originated from mesenchymal tissue, which appears most commonly in young men between the ages 10 and 30 [1]. Prior to neoadjuvant chemotherapy, amputations and disarticulations were the dominant treatments for osteosarcoma with a 5-year overall survival (OS) rate of only about 20% [2]. Currently, osteosarcoma chemotherapy mainly consists of five drugs, high-dose methotrexate (HDMTX) with leucovorin rescue, doxorubicin (adriamycin), cisplatin, ifosfamide, and etoposide. With the use of effective neoadjuvant chemotherapy in the 1970s, limb salvage surgery (LSS) has been taken as a potential treatment for osteosarcoma [3]. Usually, LSS has functional and physiological advantages over traditional amputative procedures when combined with neoadjuvant or adjuvant chemotherapy [4].

Previous studies have shown that LSS is applicative for localized osteosarcoma, while amputation is suitable for aggressive malignant osteosarcoma [5, 6]. However, there are still some surgeons holding an opposite view, considering that immediate and expanded resection of the tumor will prevent the progression of fracture-induced disease. Consequently, amputation is considered to be a better option for osteosarcoma patients with pathologic fracture [7, 8]. It has been reported that the risk of local recurrence and the 5-year OS rate did not differ significantly between LSS and amputation in osteosarcoma patients with pathological fractures [9]. Han et al. has reported that the risk of local recurrence and the 5-year OS rate did not differ significantly between LSS and amputation in osteosarcoma patients [10]. Other studies also reported a worse prognosis with osteosarcoma associated with pathologic fractures [11].

There were disputes on the survival and function recovery between treatments of LSS and amputation in patients with osteosarcoma [6, 12]. We conducted a meta-analysis on survival and function in limb osteosarcoma patients treated by LSS compared with amputation or rotationplasty [13]. In addition, our study investigated whether LSS improved survival based on 5-year rates and local cancer recurrence in osteosarcoma patients with LSS or amputation treatment [5, 14].

Through searching more abundant osteosarcoma literature, we conduct this meta-analysis to get a comprehensive conclusion in osteosarcoma patients treated by LSS and amputation. These results will help us to determine the most appropriate method to treat osteosarcoma [15].

## Methods

### Literature search

MEDLINE, Cochrane, EMBASE, and Google Scholar databases were searched for relevant data till April 30, 2016. The reference lists of relevant studies were also hand-searched. Keywords used for searching included limb surgery, salvage surgery, amputation, osteosarcoma, bone cancer, recurrence, and metastasis occurrence.

### Included studies

Studies were included if they were contrastive research between LSS and amputation groups, patients with osteosarcoma in their four limbs, and a mass of data in regard to local recurrence or 5-year overall survival rate. Exclusion standard is as follows: studies including data related to LSS or amputation groups without a comparison, case series with less than 20 total patients, letters, case reports, editorials, or reviews [16].

### Study selection and data extraction

Outcomes were collected from the articles by two authors of our study. The authors made a structured table and then collected all the data into a database. The following data were extracted from articles according to the inclusion criteria: the name of the first author, year of publication, design scheme, number of patients in each group, patients’ age and gender, local recurrence rates, and 5-year OS rates. Any disagreement was resolved by sequential discussions until an agreement was reached. Two reviewers independently assessed the quality of every included study according to the Newcastle-Ottawa Scale (NOS).

### Statistical analysis

The outcome of measurement used in our study was local recurrence and 5-year overall survival rate, which were all dichotomous data. We used the software of the Cochrane Collaboration (ReviewManager5.2) to calculate odds ratios (ORs) and 95% confidence intervals (CIs) for all outcomes. Statistical heterogeneity among the included studies was evaluated by the *I* ^2^ tests. Statistically significant heterogeneity was defined as an *I* ^2^ value >0.5 [9]. *I* ^2^ illustrates the percentage of the total variability in effect estimates among trials that is due to heterogeneity rather than to chance. A random effects model was selected for heterogeneous data; otherwise, a fixed effect model was selected. Funnel plots were used to test the possibility of publication bias, which exhibited the intervention effect from the individual study against the respective standard error. A symmetrical plot represents no bias, and any asymmetry of the plot suggests the existence of publication bias.

## Results

### Study selection and characterization

**Fig. 1:**
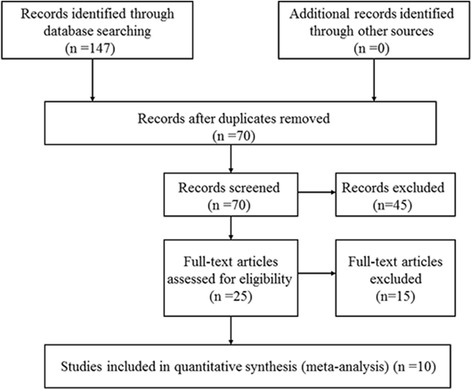
Flow chart of studies included and excluded

In the primary literature search, 147 relevant articles were retrieved and 77 were excluded based on the exclusion criteria (Fig. 1). The abstracts of the remaining 70 were screened, and 45 were excluded based on the exclusion criteria. After all the reviews of the remaining 25 studies, 15 were excluded due to lacking outcomes of interest (*n* = 10) and duplication in the study population with other articles (*n* = 5). In a word, a total of ten articles were included in the meta-analysis. Characteristics of the studies are summarized in Table 1, and outcomes are summarized in Table 2.

**Table 1:** Characteristics of the included studies

Ref.	Study period	Patient number	Male/female	Median age (range)	Enneking stage	Country
Ferrari et al.	1983–1986	127 (91/35)	72/55	15 (5–43)	Stage IIB	Austria
Bacci et al.	1986–1993	29 (25/4)	20/9	46	Stage IIB	Italy
Edmonson et al.	1976–1980	38 (1/37)	24/14	17 (9–62)	Stage IIB	Austria
Gherlinzoni et al.	1983–1988	355 (239/116)	–	–	Stage IIA	Italy
Goorin et al.	1976–1989	74 (36/38)	–	–	Stage IIA	Austria
Zhang et al.	1981–1997	31 (17/14)	26/5	15 (7–42)	Stage IIB	China
Sha et al.	1989–2006	56 (35/21)	–	18 (2–46)	Stage IIA	China
Niu et al.	1992–2001	189 (140/49)	125/64	18 (4–39)	Stage IIB	China
Bramer et al.	1983–2003	56 (44/12)	36/20	16 (3–36)	Stage IIB	America
Ferguson et al.	1989–2006	31 (19/12)	14/17	30 (11–32)	Stage IIB	America

**Table 2:** Outcomes of the included studies

Ref.	mDFS	Local recurrence (LSS/amputation)	5-year survival (LSS/amputation)	Metastatic occurrence (LSS/amputation)	Follow-up (range)
Ferrari et al.	14 (2–96)	(16/7)	(36/23)	(40/23)	130 (114–153)
Bacci et al.	22.9 (10–49)	(2/1)	(18/3)	(6/4)	96 (60–144)
Edmonson et al.	22.2 (9–64)	(0/11)	(0/20)	(1/7)	60 (31–74)
Gherlinzoni et al.	20 (5–88)	(19/13)	(67/56)	(87/67)	64 (19–88)
Goorin et al.	20 (6–67)	(17/10)	(26/29)	(21/24)	56 (7–120)
Zhang et al.	18 (9–62)	(9/6)	(11/10)	(9/7)	62 (31–71)
Sha et al.	23 (11–43)	(5/3)	(17/6)	(12/9)	55 (8–175)
Niu et al.	24 (9–64)	(21/13)	(56/34)	(35/15)	24 (32–145)
Bramer et al.	–	(6/2)	(30/8)	–	54.7 (8–146)
Ferguson et al.	–	(2/0)	(7/3)	–	–

### Local recurrence rate after surgery

**Fig. 2:**
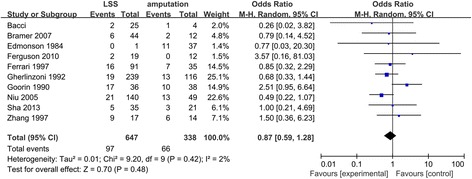
Forest plot of comparison. Local recurrence of LSS or amputation for the treatment of osteosarcoma

All the ten studies reported the local recurrence rate. Our studies reported no patients with local recurrence data for either the amputation group or the LSS group. Therefore, only ten studies with complete local recurrence rate data were included in the analysis (Fig. 2). A fixed-effects model of analysis was used [17]. There was no difference in the local recurrence rate between LSS and amputation (OR = 0.87, 95% CI 0.62–1.37, *P* = 0.42).

### 5-year overall survival and test of heterogeneity

**Fig. 3:**
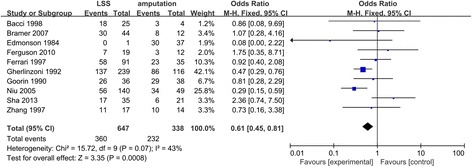
Forest plot of comparison. 5-year overall survival of LSS or amputation for the treatment of osteosarcoma

**Fig. 4:**
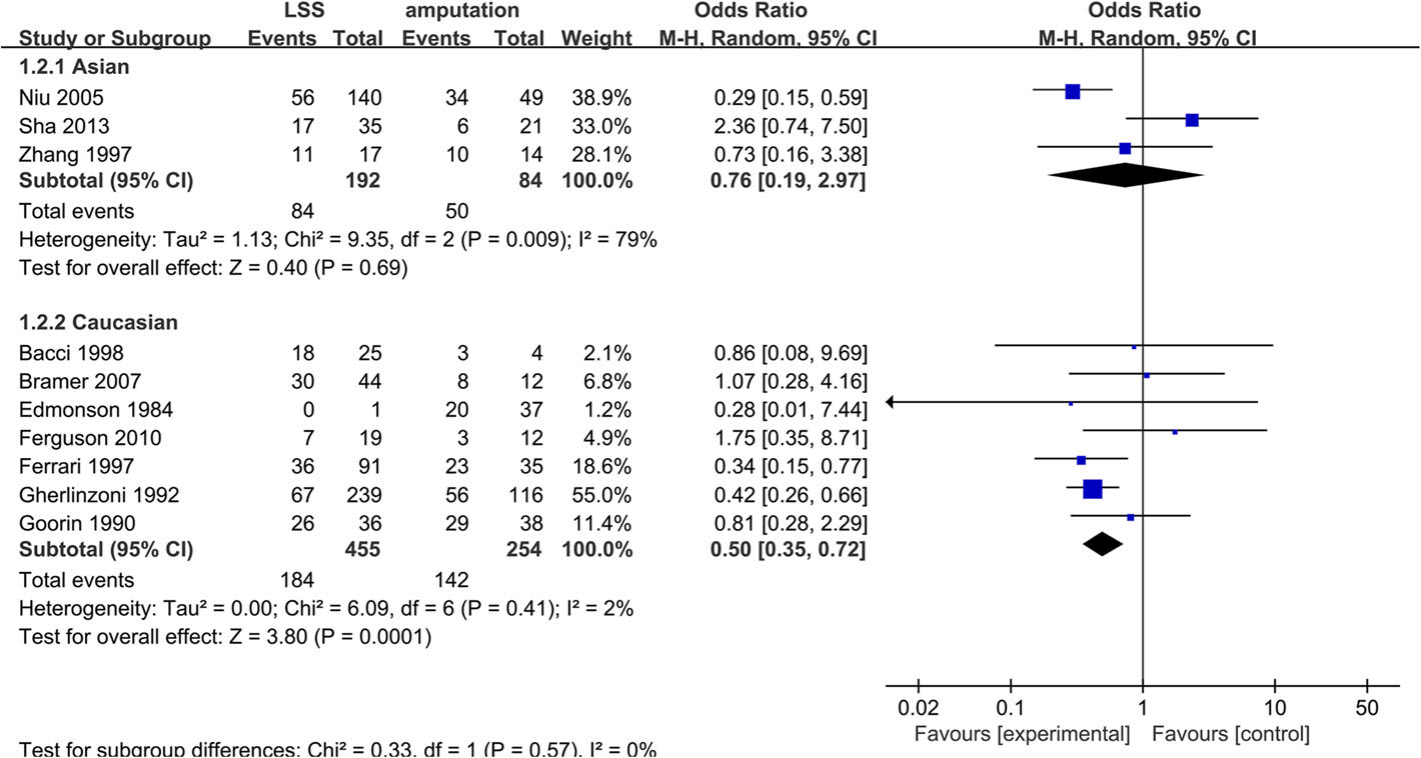
Forest plot of comparison 5-year overall survival of LSS or amputation for the treatment of osteosarcoma with excluding three studies

Among these eligible studies, a total of ten studies with 5-year survival rate data were included in the analysis (Fig. 3). Totally, there was significant heterogeneity for the comparison of 5-year overall survival between the amputation group and LSS group (*Q* test *P* value = 0.07, *I* ^2^ = 43%). According to the result of stratification analysis, we explored the source of heterogeneity from the subgroup analyses of European (Fig. 4) (*Q* test *P* value = 0.41, *I* ^2^ = 2%) and Asian people (*Q* test *P* value = 0.009, *I* ^2^ = 79%).

### Sensitivity analysis

Sensitivity analyses indicated that included studies were performed to determine the reliability of the results, with each study removed in turn [17]. The direction and magnitude of the combined estimates did not change markedly with the exclusion of individual studies, indicating that the results of the meta-analysis are reliable and suggesting that the results of this meta-analysis are stable.

### Publication bias

**Fig. 5:**
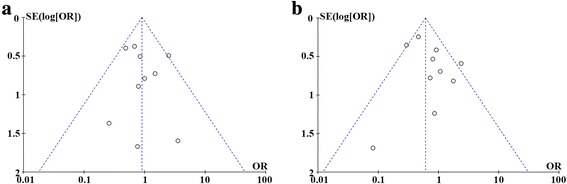
Funnel plot of comparison. **a** Local recurrence of LSS or amputation. **b** 5-year overall survival of LSS or amputation

Funnel plots of the local recurrence rates and 5-year OS rates were shown in Fig. 5. The results indicate that there is no evidence of publication bias for each of the two measures.

## Discussion

Osteosarcoma is a kind of cancerous tumor in bones [10]. Specifically, it is an aggressive malignant neoplasm that arises from primitive transformed cells of mesenchymal origin (and thus a sarcoma) and that exhibits osteoblastic differentiation and produces malignant osteoid. Osteosarcoma tends to affect regions around the knee in 60% of cases, 15% around the hip, 10% at the shoulder, and 8% in the jaw [18]. The tumor is solid, hard, and irregular due to the tumor spicules of calcified bone radiating in right angles.

Following the implementation of chemotherapy in the 1970s, the treatment of high-grade malignant osteosarcoma has made an important progress [18]. Recently, most chemotherapy regimens applied for osteosarcoma have been based around four drugs: high-dose methotrexate (HDMTX) with leucovorin rescue, doxorubicin (adriamycin), cisplatin, and ifosfamide. These agents were integrated into various chemotherapy protocols. The range of dosages most commonly used are as follows: doxorubicin (cumulative dose from 240 to 480 mg/m^2^), methotrexate (cumulative dose from 48 to 168 g/m^2^) [19, 20], cisplatin (cumulative dose from 480 to 600 mg/m^2^), and ifosfamide (cumulative dose from 30 to 69 g/m^2^) [21].

Complete surgical resection, if feasible, remains essential for cure [22]. Current surgical strategies focus on refining the nature and scope of resection to preserve uninvolved tissues. Advances in imaging techniques and positive effects of preoperative chemotherapy have led to a major shift away from amputation to limb salvage (conservative) surgery, which is expanded to limb salvage (conservative) surgery, which is expanded to around 80% of patients. Local recurrence rates of 2–3% after amputation and 5–7% after conservative surgery have been reported [22–24], while with no significant differences. The incidence of local recurrence has been closely related to the achieved surgical margins, with only a wide margin being considered appropriate. Generally, for patients who achieved complete surgical remission with adequate margins, surgical margin width in the bone did not correlate with the local recurrence rate.

With the improved efficacy of chemotherapy, the number of patients with osteosarcoma who received LSS instead of amputation has significantly increased recently. It was summarized that patients treated with LSS had a similar local recurrence compared with those treated with amputation. In the meta-analysis, we found that LSS had a similar 5-year survival compared with those treated with amputation. However, after excluding the three studies [9, 14, 25], which may be the main source of the heterogeneity and whose subjects were Asian, we found the heterogeneity reducing notably and *P* value (*P* = 0.0001) in the comparison of LSS with amputation, which proved that 5-year overall survival rate of patients treated with LSS was higher than those treated with amputation. Then in the heterogeneity test, we found that there was apparent heterogeneity among all of the eligible studies. Thus, we made a subgroup analysis, certifying that racial classification did lead to the heterogeneity. Funnel plot did not show any evidence of publication bias. Therefore, our results provide more powerful evidence to support LSS as the treatment of osteosarcoma patients.

We found that the LSS is better than amputation in functional outcomes, which are consistent with other studies [26, 27]. Johansen et al. [28] had reported notably higher functional scores after LSS compared with amputation respectively (*P* = 0.001). Another study which evaluated the long-term physical function of the patients treated with LSS found that amputees have poorer function as assessed by the MSTS score [26]. However, a study by Mei et al. [22] showed no difference in functional scores and QOL between the two types of surgical management. More clinical trials are needed to compare the functional recovery of different surgical methods.

Some limitations of this meta-analysis should be mentioned. Firstly, the lack of detailed data form in original studies made it hard to adjust estimate by age, menopausal, lifestyle, smoking, race, and so on, while more precise analysis needed this kind of adjusting. Secondly, there was no detailed data for functional recovery. Thirdly, not all control included studies were in Hardy-Weinberg equilibrium (HWE) [28].

Otherwise, our meta-analysis also has some advantages. Firstly, a systematic review of the association of survival and function in limb osteosarcoma patients with LSS or amputation treatment was statistically more powerful than any single study. Secondly, all of the case-control studies had a high quality and conformed to our inclusion criteria.

## Conclusions

In the end, our meta-analysis emphasized that LSS can improve survival of osteosarcoma patients with lower metastatic occurrence and better survival, while not increasing the risk of recurrence. Our meta-analysis also supported the hypothesis that LSS can be a new option for osteosarcoma patients.

## Abbreviations

HDMTX: High-dose methotrexate
HWE: Hardy-Weinberg equilibrium
LSS: Limb salvage surgery
NOS: Newcastle-Ottawa Scale
OS: Overall survival
